# Author Correction: High-fidelity qutrit entangling gates for superconducting circuits

**DOI:** 10.1038/s41467-023-40049-8

**Published:** 2023-07-17

**Authors:** Noah Goss, Alexis Morvan, Brian Marinelli, Bradley K. Mitchell, Long B. Nguyen, Ravi K. Naik, Larry Chen, Christian Jünger, John Mark Kreikebaum, David I. Santiago, Joel J. Wallman, Irfan Siddiqi

**Affiliations:** 1 Department of Physics, University of California, Berkeley, Berkeley, CA 94720 USA; 2 Computational Research Division, Lawrence Berkeley National Laboratory, Berkeley, CA 94720 USA; 3 Materials Science Division, Lawrence Berkeley National Laboratory, Berkeley, CA 94720 USA; 4 Keysight Technologies Canada, Kanata, ON K2K 2W5 Canada

**Keywords:** Qubits, Quantum information, Quantum mechanics

Correction to: *Nature Communications* 10.1038/s41467-022-34851-z, published online 05 December 2022

The original version of this Article contained an error in the second sentence of the Acknowledgements, which incorrectly read: ‘This work was supported by the Quantum Testbed Program of the Advanced Scientific Computing Research for Basic Energy Sciences program, Office of Science of the U.S. Department of Energy under Contract No. DE-AC02-05CH11231’. The correct version states ‘the Quantum Testbed Program of the Advanced Scientific Computing Research Division’ in place of ‘the Quantum Testbed Program of the Advanced Scientific Computing Research for Basic Energy Sciences program’. This has been corrected in both the PDF and HTML versions of the Article.

