# Supplementary material for: High-fidelity qutrit entangling gates for superconducting circuits
Source: Nat Commun. 2022 Dec 5;13:7481. doi: 10.1038/s41467-022-34851-z (PMC9722686; doi:10.1038/s41467-022-34851-z)
Supplement: Supplementary file 1 — Supplementary Information [file 41467_2022_34851_MOESM1_ESM.pdf]

Supplementary Information: High-Fidelity Qutrit Entangling Gates for Superconducting Circuits

Noah Goss,[1,2] Alexis Morvan,[2] Brian Marinelli,[1,2] Bradley K. Mitchell,[1,2] Long B. Nguyen,[2] Ravi K. Naik,[1,2]
Larry Chen,[1] Christian Jünger,[2] John Mark Kreikebaum,[1,3]
David I. Santiago,[2] Joel J. Wallman,[4] and Irfan Siddiqi[1,2,3]

[1] *Department of Physics, University of California, Berkeley, Berkeley CA 94720, USA.*
[2] *Computational Research Division, Lawrence Berkeley National Laboratory, Berkeley, California 94720, USA.*
[3] *Materials Science Division, Lawrence Berkeley National Laboratory, Berkeley, California 94720, USA.*
[4] *Keysight Technologies Canada, Kanata, ON K2K 2W5, Canada.*

(Dated: November 2, 2022)

Supplementary Note 1 . DEVICE PARAMETERS

Both gates and cross-Kerr characterization data was taken using an 8 transmon ring with fixed-frequency transmons and fixed-frequency coupling mediated by a coplanar waveguide resonator. The CZ and CZ† gate are performed on two different chips, using two different pairs of transmon qutrits. We give the relevant single-qutrit parameters and coherences for the CZ and CZ† gate in Supplementary Table 1 and Supplementary Table 2 respectively. Here, T_{2r} denotes coherence statistics taken using a Ramsey experiment and T_{2e} using an echo pulse. We additionally provide the approximate gate parameters for the two gates performed in the work, where ω_d denotes the drive frequency, Ω denotes the approximate drive strength, and τ_g denotes the total gate time including the π pulses in the $\{|1\rangle\,|2\rangle\}$ subspace.

	Q3	Q4
Qubit freq. (GHz)	5.436	5.327
Anharm. (MHz)	-260.20	-262.94
T_1^{01} (μs)	125(37)	78(16)
T_1^{12} (μs)	63(9)	47(5)
T_{2e}^{01} (μs)	190(28)	138(25)
T_{2e}^{12} (μs)	61(13)	45(7)
T_{2e}^{02} (μs)	75(19)	62(6)
T_{2r}^{01} (μs)	114(47)	99(24)
T_{2r}^{12} (μs)	17(8)	17(9)
T_{2r}^{02} (μs)	20(16)	21(9)
ω_d (GHz)	5.287	5.287
Ω (MHz)	≈ 13	≈ 13
τ_g (ns)	783	

Supplementary Table 1. Single-qutrit parameters for the pair of transmons used to perform the CZ gate.

	Q5	Q6
Qubit freq. (GHz)	5.362	5.523
Anharm. (MHz)	-275	-271.35
T_1^{01} (μs)	45(7)	58(7)
T_1^{12} (μs)	33(3)	28(3)
T_{2e}^{01} (μs)	63(7)	84(6)
T_{2e}^{12} (μs)	28(3)	30(3)
T_{2e}^{02} (μs)	37(3)	35(3)
T_{2r}^{01} (μs)	36(9)	76(8)
T_{2r}^{12} (μs)	10(6)	18(6)
T_{2r}^{02} (μs)	11(6)	21(8)
ω_d (GHz)	5.191	5.191
Ω (MHz)	≈ 11	≈ 11
τ_g (ns)	580	

Supplementary Table 2. Single-qutrit parameters for the pair of transmons used to perform the $\mathrm{CZ}^\dagger$ gate.

Supplementary Note 2 . PERTURBATION THEORY

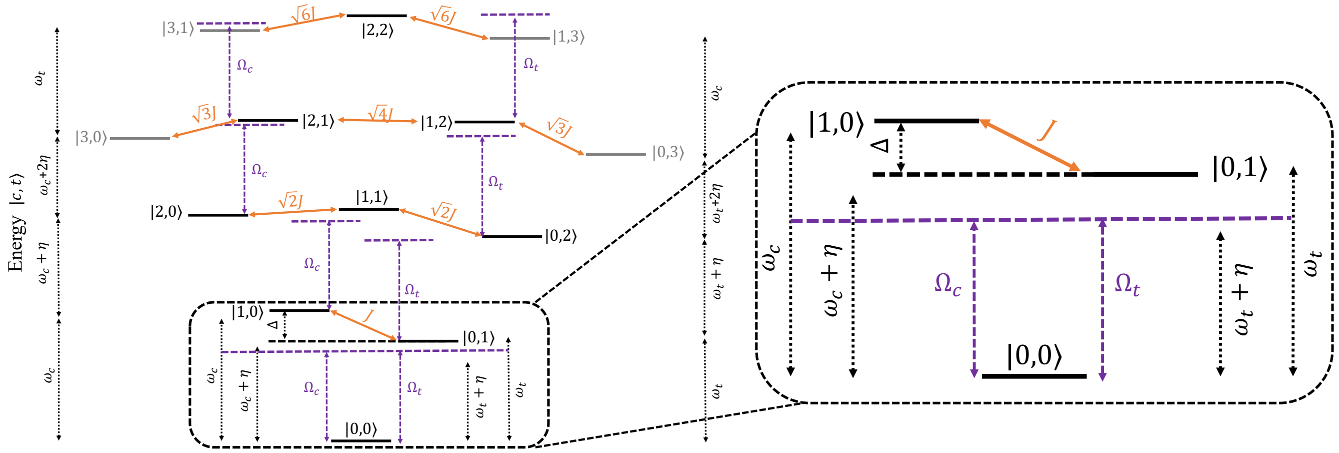

Supplementary Fig. 1. **Drive scheme for conditional stark-induced cross-Kerr Hamiltonian** . We present an example energy level diagram in the rotating frame of the drive where we place the off resonant microwave drive on both qutrits between their two respective $|1\rangle \to |2\rangle$ transitions. The simultaneous off resonant microwave drives induce conditional Stark shifts that generate entangling phases on four states in our two qutrit Hilbert space.

In the frame of the drive at frequency ω_{d} and after making a rotating wave approximation (RWA), the system Hamiltonian is

$$H = \sum_{i=\mathrm{c,t}} \left[(\omega_i - \omega_{\mathrm{d}})\, a_i^\dagger a_i + \frac{\eta_i}{2} a_i^\dagger a_i^\dagger a_i a_i + \Omega_i \left(e^{i\varphi_i} a_i + e^{-i\varphi_i} a_i^\dagger \right) \right] + J \left(a_{\mathrm{c}}^\dagger a_{\mathrm{t}} + a_{\mathrm{c}} a_{\mathrm{t}}^\dagger \right) \tag{S1}$$

with $\hbar = 1$ and the transmons approximated as Duffing oscillators with qubit frequency ω_i, anharmonicity η_i, capacitive coupling J, and where we define a_i as the bosonic annihilation operator. The parameters of the drive are given as amplitude Ω_i, and phase φ_i. Since only the relative drive phase is physical, we choose a basis where $\varphi_{\mathrm{c}} = 0$ and $\varphi_d = \varphi_t - \varphi_c$. The detuning of transmon i from the drive is $\Delta_i = \omega_i - \omega_d$. We analyze the system perturbatively in the limit $\Omega_i, J \ll |\eta_i|, |\Delta_i|$. In this limit the bare transmon Hamiltonians serve as the unperturbed system, H_0, and the perturbation, V, is composed of the single qubit drive terms and the coupling term. Time independent perturbation theory applied to Eq. S1 will yield energies E_{ij}, the approximate diagonal elements of the Hamiltonian in the basis labelled by the transmon occupation numbers $|ij\rangle$ with $i, j = 0, 1, 2, \ldots, d-1$ the state of the "control" (c) and "target" (t) transmons respectively. The dimension where we truncate the Hamiltonian is d. In the present case of qutrits $d = 3$ and $H' \approx \sum_{i,j=0}^{2} \tilde{E}_{ij} |ij\rangle \langle ij|$ where we define $\tilde{E}_{ij} = E_{ij} - E_{00}$, performing a global shift of the energies to set the energy of the $|00\rangle$ state to zero.

We isolate the entangling cross-Kerr terms by transforming H' according to the unitary

$$U = \exp\Big\{ -it \left[\tilde{E}_{01} I \otimes |1\rangle \langle 1| + \tilde{E}_{10} |1\rangle \langle 1| \otimes I + \tilde{E}_{02} I \otimes |2\rangle \langle 2| + \tilde{E}_{20} |2\rangle \langle 2| \otimes I \right] \Big\}, \tag{S2}$$

where $I = |0\rangle \langle 0| + |1\rangle \langle 1| + |2\rangle \langle 2|$ is the single qutrit identity operator. The transformation eliminates the single qutrit energies $\tilde{E}_{i0}$ and $\tilde{E}_{0j}$, $i, j = 1, 2$ which simply result in local phases that can be eliminated by virtual single qutrit phase gates. The transformed Hamiltonian

$$H'' = \alpha_{11} |11\rangle \langle 11| + \alpha_{21} |21\rangle \langle 21| + \alpha_{12} |12\rangle \langle 12| + \alpha_{22} |22\rangle \langle 22| \tag{S3}$$

is written in terms of the cross-Kerr rates α_{ij} which describe the rates at which the entangling phases on the states $|ij\rangle$ are accumulated. These α_{ij}, explicitly given by

$$\alpha_{ij} = E_{ij} + E_{00} - E_{0j} - E_{i0}, \tag{S4}$$

generalize the qubit ZZ rate ζ to the case of qutrits [1].

In perturbation theory we expand $\alpha_{ij} = \alpha_{ij}^{(0)} + \alpha_{ij}^{(1)} + \cdots$ where $\alpha_{ij}^{(n)} = E_{ij}^{(n)} + E_{00}^{(n)} - (E_{i0}^{(n)} + E_{0j}^{(n)})$ and $E_{ij}^{(n)}$ is the nth order correction to the energy of state $|ij\rangle$. The first two terms $\alpha_{ij}^{(0)}$ and $\alpha_{ij}^{(1)}$ are zero because in the unperturbed system, H_0, the transmons are not interacting and the perturbation V only maps states $|ij\rangle$ to states $|i'j'\rangle$ that are orthogonal to $|ij\rangle$. The first contribution comes at second order where the static coupling of strength J between the transmons generates a static cross-Kerr interaction with rates

$$\alpha_{11}^{(2)} = \frac{4\eta J^2}{(\eta-\Delta)(\eta+\Delta)} \tag{S5}$$

$$\alpha_{21}^{(2)} = \frac{2\eta J^2(5\eta-4\Delta)}{\Delta(\eta-\Delta)(2\eta-\Delta)} \tag{S6}$$

$$\alpha_{12}^{(2)} = -\frac{2\eta J^2(5\eta+4\Delta)}{\Delta(\eta+\Delta)(2\eta+\Delta)} \tag{S7}$$

$$\alpha_{22}^{(2)} = \frac{16\eta J^2}{(\eta+\Delta)(\eta-\Delta)} \tag{S8}$$

where $\Delta = \omega_{\mathrm{c}} - \omega_{\mathrm{t}}$ and we have set $\eta_{\mathrm{c}} = \eta_{\mathrm{t}} = \eta$ in order to arrive at more compact expressions. In most systems this is a reasonable approximation and in particular the pair of transmons used in this work have anharmonicities $\eta_{\mathrm{c}} = 272$ MHz and $\eta_{\mathrm{t}} = 270$ MHz.

As in the qubit case the driven cross-Kerr interaction contributes starting at third order when both transmons are driven (see the discussion in the Supplementary Materials of [1]) and we find rates

$$\alpha_{11} = \frac{8\cos\phi\,\eta^2\Omega_C\Omega_T J}{\Delta_C\Delta_T(\eta-\Delta_C)(\eta-\Delta_T)} \tag{S9}$$

$$\alpha_{12}/\alpha_{11} = \frac{\Delta_C^2(\eta-2\Delta_T)+\eta(2\eta^2-2\eta\Delta_T-\Delta_T^2))+\Delta_C(-3\eta^2+2\eta\Delta_T+2\Delta_T^2)}{(2\eta-\Delta_C)(2\eta-\Delta_T)} \tag{S10}$$

$$\alpha_{21}/\alpha_{11} = \frac{\Delta_T^2(\eta-2\Delta_C)+\eta(2\eta^2-2\eta\Delta_C-\Delta_C^2)+\Delta_T(-3\eta^2+2\eta\Delta_C+2\Delta_C^2)}{(2\eta-\Delta_C)(2\eta-\Delta_T)} \tag{S11}$$

$$\alpha_{22}/\alpha_{11} = \frac{(\eta-2\Delta_C)(\eta-2\Delta_T)}{(2\eta-\Delta_C)(2\eta-\Delta_T)} \tag{S12}$$

The expressions are complicated but we now point out some important features. In typical systems the drive strengths Ω_i are larger than the coupling strength J and as a result $\alpha_{ij}^{(3)} > \alpha_{ij}^{(2)}$. For example, the CZ gate described in the main text is performed with $\Omega_{\mathrm{c}}, \Omega_{\mathrm{t}} \approx 11$ MHz on a pair of transmons with estimated coupling $J = 2.7$ MHz. Therefore, the static cross-Kerr can in principle be cancelled by the driven cross-Kerr. The result also gives the leading order linear dependence of the rates α_{ij} on the drive strengths Ω_i and the coupling J as well as their sinusoidal dependence on the relative drive phase φ (see Figure 2 in the main text).

Supplementary Note 3 . THE WEYL AND GELL-MANN BASES

When analyzing qubits, we use tensor products of the single-qubit Pauli operators $\mathbb{P} = \{I, X, Y, Z\}$. These operators have the following helpful properties:

1. they form a projective group under matrix multiplication;
2. they are unitary;
3. they are a trace-orthogonal basis for the space of operators; and
4. they correspond to natural Hamiltonians.

Unfortunately, no set of operators with the same properties exist for higher dimensions. We thus need to separate some of the properties when analyzing qudits.

There are two sets of operators, namely, the Weyl and Gell-Mann operators that, taken together, satisfy all four properties and also coincide with the Pauli operators in 2D. We now review these two sets of operators.

The Gell-Mann operators are obtained by embedding the familiar Pauli matrices into two-dimensional subspaces of a higher dimensional space. Recall that the standard single-qubit Pauli operators are

$$\begin{aligned} X &= |1\rangle\langle 0| + |0\rangle\langle 1| \\ Y &= i\,|1\rangle\langle 0| - i\,|0\rangle\langle 1| \\ Z &= |0\rangle\langle 0| - |1\rangle\langle 1|\,. \end{aligned} \tag{S13}$$

We can embed these operators in a d-dimensional subspace by defining

$$\begin{aligned} X^{jk} &= |j\rangle\langle k| + |k\rangle\langle j| \\ Y^{jk} &= i\,|j\rangle\langle k| - i\,|k\rangle\langle j| \\ Z^{jk} &= |j\rangle\langle j| - |k\rangle\langle k| \end{aligned} \tag{S14}$$

for $0 \leq j < k < d$. The X^{jk} and Y^{jk} operators are trace-orthogonal Hermitian operators and also correspond to transitions between two levels, which is the natural way to control a harmonic oscillator. Specifically, we can drive the X^{01} and X^{12} Hamiltonians by applying tones at ω_{01} and ω_{12}, which generate Rabi oscillations in the corresponding qubit subspaces of the qutrit, from which we can generate the single qutrit gates

$$X^{01}_{\pi/2} = \frac{1}{\sqrt{2}}\begin{pmatrix} 1 & -i & 0 \\ -i & 1 & 0 \\ 0 & 0 & 1 \end{pmatrix}, \quad X^{12}_{\pi/2} = \frac{1}{\sqrt{2}}\begin{pmatrix} 1 & 0 & 0 \\ 0 & 1 & -i \\ 0 & -i & 1 \end{pmatrix}. \tag{S15}$$

Similarly, we can perform virtual Z gates within these two qubit subspaces of the qutrit, which natively yield the continuous gates

$$Z^{01}(\phi) = \begin{pmatrix} e^{-i\phi} & 0 & 0 \\ 0 & 1 & 0 \\ 0 & 0 & 1 \end{pmatrix}, \quad Z^{12}(\phi) = \begin{pmatrix} 1 & 0 & 0 \\ 0 & 1 & 0 \\ 0 & 0 & e^{i\phi} \end{pmatrix} \tag{S16}$$

From these four gates, $X^{01}_{\pi/2}, X^{12}_{\pi/2}, Z^{01}(\phi), Z^{12}(\phi)$, we can compile an arbitrary unitary in $\mathbb{U}(3)$ using at most 6 of these gates, with a decomposition given in Ref [2].

However, while the Z^{jk} are Hermitian operators, they are not linearly independent and so do not provide a suitable basis for quantum process tomography. We thus extend the set $\{X^{jk}, Y^{jk} : 0 \leq j < k < d\}$ to a trace-orthogonal basis by adding a trace-orthogonal set of diagonal operators. A natural choice would be the projectors onto the

computational basis, however, this obscures the fact that all density operators have unit trace. We thus use the operators

$$D_j = -j\,|j\rangle\langle j| + \sum_{0\le k<j} |k\rangle\langle k| \tag{S17}$$

for $1 \le j < d$, together with the identity operator to obtain the Gell-Mann basis. Here the Gell-Mann matrices λ_i plus the identity I_3 span SU(3) and are a natural choice for qutrit Pauli transfer matrices (PTMs). For convenience, we index these elements for a single qutrit as follows:

$$\begin{gathered}\mathrm{I}_3 = \begin{pmatrix}1&0&0\\0&1&0\\0&0&1\end{pmatrix}, \lambda_1 = \begin{pmatrix}0&1&0\\1&0&0\\0&0&0\end{pmatrix}, \lambda_2 = \begin{pmatrix}0&-i&0\\i&0&0\\0&0&0\end{pmatrix}, \lambda_3 = \begin{pmatrix}1&0&0\\0&-1&0\\0&0&0\end{pmatrix}, \lambda_4 = \begin{pmatrix}0&0&1\\0&0&0\\1&0&0\end{pmatrix},\\ \lambda_5 = \begin{pmatrix}0&0&-i\\0&0&0\\i&0&0\end{pmatrix}, \lambda_6 = \begin{pmatrix}0&0&0\\0&0&1\\0&1&0\end{pmatrix}, \lambda_7 = \begin{pmatrix}0&0&0\\0&0&-i\\0&i&0\end{pmatrix}, \lambda_8 = \frac{1}{\sqrt{3}}\begin{pmatrix}1&0&0\\0&1&0\\0&0&-2\end{pmatrix}\end{gathered} \tag{S18}$$

Having defined the Gell-Mann basis, we now define the Heisenberg-Weyl operators, which are a unitary generalization of the familiar single-qubit Pauli operators to higher dimensional spaces (qudits) that enable the Clifford group to be generalized. Let d be a positive integer and $\mathbb{Z}_d = \{0, \ldots, d-1\}$ denote the set of integers modulo d. The generalizations of the X and Z operators to qudits are

$$\begin{aligned} X &= \sum_{j\in\mathbb{Z}_d} |j \oplus_d 1\rangle\langle j| \\ Z &= \sum_{j\in\mathbb{Z}_d} \exp\left(\frac{2\pi i}{d} j\right) |j\rangle\langle j|\,, \end{aligned} \tag{S19}$$

where $\oplus_d$ denotes addition modulo d. From this, the definition of the two-qudit generalization of a controlled-Z gate (CZ) follows naturally as:

$$U_{CZ} = \sum_{n=1}^{d} |n\rangle\langle n| \otimes Z^n \tag{S20}$$

For the two qutrit entangling gates performed in this work, this yields the follow unitaries:

$$U_{CZ} = e^{2\pi i/3}(|11\rangle\langle 11| + |22\rangle\langle 22|) + e^{4\pi i/3}(|12\rangle\langle 12| + |21\rangle\langle 21|) + \sum_{j=0}^{2}(|0j\rangle\langle 0j| + |j0\rangle\langle j0|) \tag{S21}$$

$$U_{CZ^\dagger} = e^{4\pi i/3}(|11\rangle\langle 11| + |22\rangle\langle 22|) + e^{2\pi i/3}(|12\rangle\langle 12| + |21\rangle\langle 21|) + \sum_{j=0}^{2}(|0j\rangle\langle 0j| + |j0\rangle\langle j0|) \tag{S22}$$

The Weyl basis for $\mathbb{C}^{d\times d}$ is the set

$$\mathbb{W}_d = \{W_{xz} = X^x Z^z : x, z \in \mathbb{Z}_d\}\,, \tag{S23}$$

which is a trace-orthogonal basis for $\mathbb{C}^{d\times d}$ as it satisfies

$$\operatorname{tr} W^\dagger V = d\delta_{W,V} \quad \forall\, W, V \in \mathbb{W}_d. \tag{S24}$$

Let n be a positive integer and $D = d^n$. Then we define the n-qudit Weyl basis to be the set $\mathbb{W}_{d,n} = \mathbb{W}_d^{\otimes n}$, which is a trace-orthogonal basis for $\mathbb{C}^{D\times D}$. Note that the Weyl basis is *not* a proper group as it is not closed under

multiplication. However, every element of the closure of the Weyl basis is proportional to an element of the Weyl basis up to an overall phase. This overall phase vanishes in all cases as we only consider the adjoint action of Weyl operators and so we treat the Weyl basis as a projective group. The n-qutrit Clifford group is then defined to be the normalizer of the extended Weyl group $\mathbb{EW}_{d,n} = \mathbb{U}(1)\mathbb{W}_{d,n}$ (which is a proper group), that is,

$$\mathfrak{C}_{d,n} = \{U \in \mathbb{U}(d^n) : U\mathbb{EW}_{d,n}U^\dagger = \mathbb{EW}_{d,n}\}. \tag{S25}$$

Supplementary Note 4 . ASSUMPTIONS FOR CHARACTERIZATION

We now define the assumption of time-dependent Markovian noise that we use to analyze the results of our characterization protocols. At a high level, we assume that each operation applied to the system corresponds to a linear map that is independent of what other maps have been applied but may depend on the number of operations that have been applied since the system was initialized. We allow this time dependence primarily to facilitate post-processing of time-stationary noise processes wherein the physical process does not depend on the number of operations that have been applied since the system was initialized but we are performing a weighted average over the operation that is applied in the jth time step.

Formally, for a vector space $\mathbb{V}$ let $\mathbb{V}^*$ denote the dual of $\mathbb{V}$ and $\mathcal{L}(\mathbb{V})$ denote the set of linear maps from $\mathbb{V}$ to itself. Then we assume the following.

1. The state space of a physical implementation of an n-qudit system is some fixed vector space $\mathbb{V}$.
2. Preparing the system in the state ρ corresponds to setting the state of the quantum system to some $\Theta_\rho \in \mathbb{V}$.
3. Applying some unitary operation U to the system in the jth time step after preparing the system in a state corresponds to applying some linear map, $\Theta(j, U) \in \mathcal{L}(\mathbb{V})$ to the state of the system.
4. The expectation value of an observable Q is obtained by applying some fixed $\Theta_Q \in \mathbb{V}^*$. (Note that we will ignore finite measurement statistics for now.)

With some abuse of notation, we refer to the functions $\Theta_j : \mathbb{N} \times \mathbb{U}(d^n) \to \mathcal{L}(\mathbb{V} \to \mathbb{V})$ and the vectors Θ_ρ and Θ_Q together as the implementation map Θ [3].

The assumption of time-dependent Markovian noise allows hidden Markovianity as the implementation map can include a coupling to an environment. This hidden Markovianity is frequently referred to as non-Markovianity in the quantum information community. However, we allow it in the general setting because the additional assumptions (such as positivity and complete positivity) are more cumbersome to define and typically are only helpful in the final steps of an analysis. Indeed, it is conceptually useful to include post-processing steps that depend only on one time step into an "effective" implementation map.

An ideal isolated implementation is one wherein there is a linear isomorphism between $\mathbb{V}$ and $\mathbb{C}^{D\times D}$. For concreteness, we define an isomorphism $|*\rangle\rangle_{\mathbb{B}} : \mathbb{C}^{D\times D} \to \mathbb{V}$ relative to a trace-orthonormal basis $\mathbb{B} \subset \mathbb{C}^{D\times D}$ as follows. As $\mathbb{B}$ is a trace-orthonormal basis, we can write any $A \in \mathbb{C}^{D\times D}$ in terms of $\mathbb{B}$ as

$$A = \sum_{B\in\mathbb{B}} \mathrm{tr}\big(B^\dagger A\big) B. \tag{S26}$$

Therefore we can choose $\{|B\rangle\rangle_{\mathbb{B}} : B \in \mathbb{B}\}$ to be an orthonormal basis of $\mathbb{V}$ and extend it to an isomorphism by defining

$$|A\rangle\rangle_{\mathbb{B}} = \sum_{B\in\mathbb{B}} \mathrm{tr}\big(B^\dagger A\big) |B\rangle\rangle_{\mathbb{B}}. \tag{S27}$$

We will typically suppress the subscript $\mathbb{B}$ as it will be clear from the context (either the normalized Weyl basis or the normalized Gell-Mann basis). To avoid having to define normalized versions of the bases explicitly, we define

$$\hat{B} = B/\sqrt{\mathrm{tr}\, B^\dagger B}. \tag{S28}$$

Moreover, defining $\langle\langle A| = |A\rangle\rangle^\dagger$ and $\langle\langle A|B\rangle\rangle = \langle\langle A||B\rangle\rangle$ and using the fact that $\mathbb{B}$ is a trace-orthonormal basis and $\{|B\rangle\rangle : B \in \mathbb{B}\}$ is an orthonormal basis, we have

$$\begin{aligned} \mathrm{tr}\, A^\dagger T &= \sum_{B,C\in\mathbb{B}} \mathrm{tr}\big(A^\dagger B\big)\, \mathrm{tr}\big(C^\dagger T\big)\, \mathrm{tr}\big(B^\dagger C\big) \\ &= \sum_{B\in\mathbb{B}} \mathrm{tr}\big(A^\dagger B\big)\, \mathrm{tr}\big(B^\dagger T\big) \\ &= \langle\langle A|T\rangle\rangle. \end{aligned} \tag{S29}$$

With the above isomorphism, we can define the ideal implementation of a unitary operator $U \in \mathbb{U}(D)$ to be

$$\phi_{\mathbb{B}}(U) = \sum_{B \in \mathbb{B}} |UBU^\dagger\rangle\rangle\langle\langle B|, \tag{S30}$$

which, by linearity, will satisfy

$$\phi_{\mathbb{B}}(U)|A\rangle\rangle = |UAU^\dagger\rangle\rangle. \tag{S31}$$

As above, we will also suppress the $\mathbb{B}$ on ϕ when the basis is clear from the context. Moreover, as $U\mathbb{B}U^\dagger$ is a trace-orthonormal basis, one can readily verify that $\phi(U)\phi(V) = \phi(UV)$ for all $U, V \in \mathbb{U}(D)$, that is, ϕ is a representation of $\mathbb{U}(D)$.

Supplementary Note 5 . CROSS ENTROPY BENCHMARKING

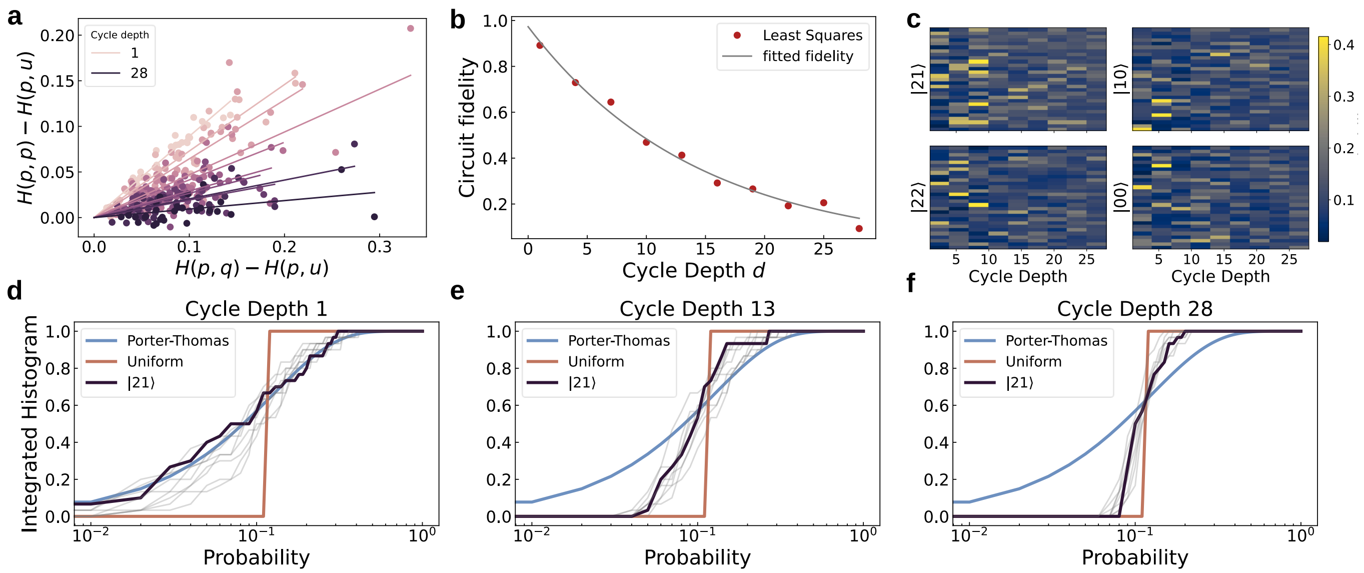

Supplementary Fig. 2. **Cross entropy benchmarking (XEB) for qutrit**, **a**, For the XEB circuits, the entropy difference $H(p,q) - H(p,u)$ is plotted against the ideal $H(p,p) - H(p,u)$ at each cycle depth, with 30 randomizations at each depth. The linear fit gives the fidelity at that particular depth. **b**, We show the exponential decay of the depolarized CZ† fidelity obtained from the linear fits in a. **c**, We plot an example of the speckle purity decay as a function of cycle depth for a representative set of four states in the two-qutrit Hilbert space. The probabilities of measuring the given tritstring are shown as a function of cycle depth across all 30 randomizations. The bright "speckle" pattern characteristic of the Porter-Thomas distribution at low circuit depths is smoothed out at larger depths. **d**-**f**, We show that the distribution of tritstrings transitions from the Porter-Thomas ditribution at low depth, to a uniform distribution at deeper depths. The CDF is emphasized for a representative tritstring, $|21\rangle$, while the CDFs of the other tritstrings are shown in grey to show typical variation of the CDF across different states.

As discussed in the main text, performing randomized benchmarking (RB) on a two qutrit gate is prohibitively expensive [2]. We utilize cross entropy benchmarking (XEB) as a second SPAM (state preparation and measurement) free benchmarking protocol to corroborate the fidelity obtained from cycle benchmarking (CB). Recently XEB played a central role in the quantum supremacy experiment in [4] and was used to benchmark the non-Clifford, three qubit iToffoli gate in [5]. XEB theory has been discussed in detail in previous works so we present only a brief review of the method before outlining more explicitly how it can be used to benchmark the two-qutrit CZ gate demonstrated here.

For XEB purposes a convenient definition of a random quantum circuit (RQC) is a quantum circuit randomly selected from an ensemble of circuits such that the distribution of probabilities (across the ensemble of circuits) of observing a particular ditstring, x, follows the Porter-Thomas distribution (for all possible ditstrings x) [6]. In the present case of quantum circuits involving two qutrits we consider the probabilities of tritstrings 00, 01, …, 21, 22. After averaging over an ensemble of random circuits errors are tailored to be purely depolarizing so an error corresponds to the circuit outputting a fully mixed state. In a mixed state the tritstring distribution is uniform with each outcome x_i having equal probability of $1/3^n$ for an n qutrit system. Thus, intuitively the circuit fidelity (under this error model) can be thought of as the deviation of the measured tritstring distribution from the uniform distribution. The XEB fidelity makes this relationship precise as we now outline.

Denote the possible tritstrings x_i for $i = 1, \dots, 3^n$ and let $p(x_i)$ be the ideal tritstring distribution for the output of a particular quantum circuit. Then $q(x_i)$ is the measured distribution. The linear cross entropy of two probability

distributions $p_1(x)$ and $p_2(x)$ with the same support is defined as

$$H(p_1, p_2) = \sum_x p_1(x) p_2(x) \tag{S32}$$

where the sum runs over the full support of the probability distributions and the self entropy is written compactly as $H(p_1) \equiv H(p_1, p_1)$. Under the depolarizing error model it can be shown straightforwardly that the circuit fidelity is

$$F_{\mathrm{XEB}} = \frac{H(p,q) - H(p,u)}{H(p,p) - H(p,u)} \tag{S33}$$

where $u(x_i)$ is the uniform probability distribution [4]. This is ultimately the difference in the ideal to measured and ideal to uniform cross entropies, normalized by the difference if the measured distribution were to perfectly match the ideal distribution ($p(x_i) = q(x_i)$ for all i).

We follow the XEB protocol for benchmarking gates/cycles outlined in Ref. [4]. This consists of measuring two qutrit circuits of varying cycle depths M where cycle i consists of two single qutrit gates $G_{i,j}$ where $i = 1, \ldots, M$ labels the cycle and $j = 1, 2$ labels the qutrit, followed by the two qutrit CZ gate† (see Supplementary Fig. 2a). The single qutrit gates are randomly selected unitaries from $SU(3)$ and decomposed according to the decomposition given in [7]. For each cycle depth M we generate N random circuits to be run and compute the ideal probability distributions of the output tritstrings for each circuit. The XEB fidelity at cycle depth M, $F_{\mathrm{XEB},M}$ is determined by performing a least squares fit of the linear relationship between $H(p_i, q_i) - H(p_i, u)$ and $H(p_i, p_i) - H(p_i, u)$ with $i = 1, \ldots, N$ labeling the random circuit at the given depth (see Figure). The cycle infidelity, $\epsilon_{\mathrm{cycle}}$, is estimated from the exponential decay of $F_{\mathrm{XEB},M}$ as a function of cycle depth M. The error rate extracted in this way agrees well with CB (see Benchmarking section in the main text).

Next we validate our implementation of XEB by verifying that the distribution of probabilities of a given tritstring across the ensemble of random circuits does indeed approach the Porter-Thomas distribution,

$$\mathcal{P}(p) = (D-1)(1-p)^{D-2} \approx De^{-Dp} \tag{S34}$$

where again D is the Hilbert space dimension, $D = 3^2$ for two qutrits and the approximate equality holds in the limit of large Hilbert space dimension, $D \gg 1$. Next we consider the evolution of this distribution as a function of cycle depth. At short to intermediate cycle depths, the circuit is sufficiently random and the distribution approaches Porter-Thomas. At larger cycle depths, the distribution begins to converge to $\mathcal{P}(p) \to \delta(p - 1/D)$ since the depolarizing errors dominate and the tritstring distributions approach the uniform distribution for all circuits. We plot our experimental observation of this behavior in Fig. 2d-f.

The method of Speckle Purity Benchmarking (SPB) is based on this observation. Denoting the state purity as γ, it can be estimated at depth M from the raw results of the XEB protocol outlined above by the relationship

$$\gamma(M) = \mathrm{Var}(p_M) \frac{D^2(D+1)}{(D-1)} \tag{S35}$$

where p_M is the set of measured probabilities of a given tritstring x across the N random circuits at cycle depth M [4]. Thus, once we have demonstrated that the distribution does indeed converge to the Porter-Thomas distribution we can estimate the decay of the state purity from just the variance of the distribution, without the need for state tomography which requires an exponential number of measurements, $3^{n(d-1)}$ where n is the number of qudits and d the dimension of each qudit. For our present case of two qutrits we would need to perform 81 measurements per circuit to determine the state purity by state tomography.

Supplementary Note 6 . EXTRACTING AN ERROR BUDGET ESTIMATION FROM CYCLE BENCHMARKING RESULTS

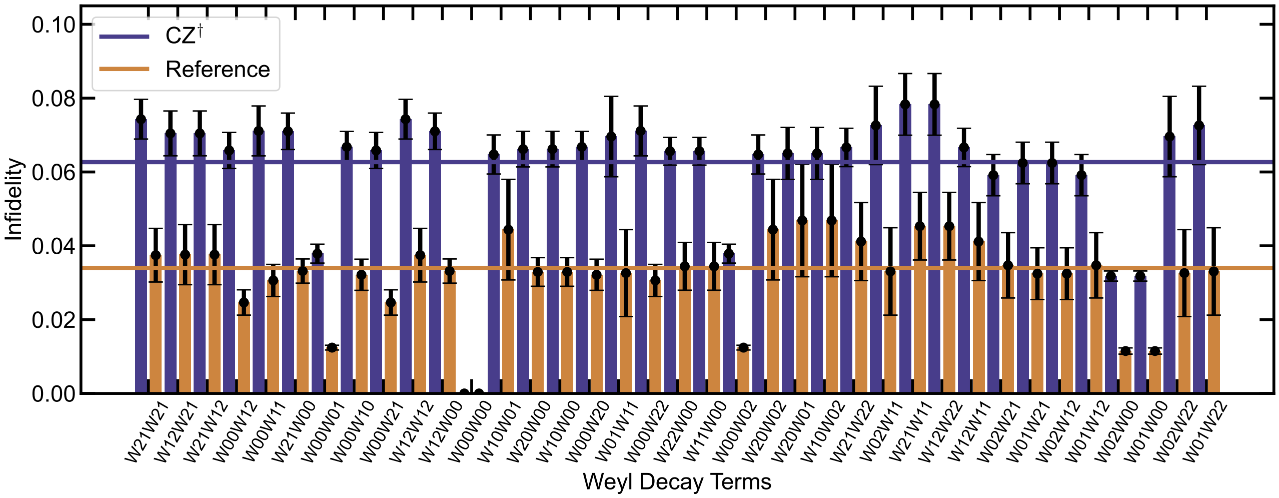

Supplementary Fig. 3. **Cycle benchmarking results with Weyl decay terms of CZ† gate**. The cycle benchmarking results shown analyze a subset of 53 Weyl channels at depths $m \in \{0, 3, 6, 15\}$. In order to provide a SPAM free estimation of the error, we compare our CB results to a reference CB experiment without the two qutrit gate to isolate and subtract the error due to the Weyl twirling itself. Here we plot only the shared decay channels analyzed for both the CZ† and reference CB experiments. The error bars are due to the uncertainty in the fitting of each decay.

We now outline how data from cycle benchmarking [8] can be used to break down the error budget for a multi-qudit Clifford gate. The following analysis also generalizes that of [8] to gate-dependent noise and allows some non-Markovian errors (namely, couplings to an environment).

A. The cycle benchmarking protocol

We now outline the cycle benchmarking protocol. Let d, m, and n be positive integers, ψ be a quantum state, and Q be an observable. Then a cycle benchmarking circuit consists of the following:

1. Prepare the system in a state ψ;
2. For each $j \in \mathbb{Z}_m$,
   (a) Choose an n-qudit Weyl operator $W_j \in \mathbb{W}_d^{\otimes n}$ uniformly at random;
   (b) Apply W_j to the system; and
   (c) Apply C to the system.
3. Choose an n-qudit Weyl operator $W_m \in \mathbb{W}_d^{\otimes n}$ uniformly at random;
4. Apply W_m to the system; and
5. Measure the expectation value of an operator Q.

For time-dependent Markovian noise, the expectation value for the cycle benchmarking protocol for a fixed choice of random Weyl operators $\vec{W} = (W_0, \ldots, W_m)$, preparation ψ and operator Q relative to an implementation map Θ is

$$\mu_{\Theta,\psi,\vec{W},Q} = \Theta_Q \left(\prod_{j=m\to 1} \Theta(2j, W_j)\Theta(2j-1, C) \right) \Theta(0, W_0)\Theta_\psi. \tag{S36}$$

The Weyl operators are sampled uniformly and independently and are uncorrelated in eq. (S36). The average over all choices of random Weyl operators is then

$$\mathbb{E}_{\vec{W}}\mu_{\Theta,\psi,\vec{W},Q} = \Theta_Q \left(\prod_{j=m\to 1} \Theta(j)\Theta(2j-1, C) \right) \Theta(0)\Theta_\psi\,, \tag{S37}$$

where for an implementation map Θ we define

$$\Theta(j) = \mathbb{E}_{W\in\mathbb{W}_{d,n}}\Theta(2j, W). \tag{S38}$$

That is, we have factorized the average of the expectation values into a product of independent averages.

B. CB decay

Before turning to analyze the variance, we first show how the above method is equivalent to that of Ref. [8] under equivalent assumptions. First, let Λ denote the physical implementation map and assume that for all $W \in \mathbb{W}_{d,n}$, we have

$$\Lambda(W) = \mathcal{L}\phi(W)\mathcal{R} \tag{S39}$$

for some linear maps $\mathcal{L}$ and $\mathcal{R}$. We then redefine

$$\begin{aligned} \Lambda_\psi &\to \mathcal{R}\Lambda_\psi \\ \Lambda(2j-1, C) &\to \mathcal{R}\Lambda(2j-1, C)\mathcal{L}, \\ \Lambda_Q &\to \Lambda_Q\mathcal{L}, \end{aligned} \tag{S40}$$

so that without loss of generality we can set $\mathcal{L}$ and $\mathcal{R}$ to the identity, that is, we can assume that the implementation of the random operations is effectively ideal.

One can readily verify that the ideal implementation of a Weyl operator W satisfies

$$\phi(W) = \sum_{V\in\mathbb{W}_{d,n}} |W\hat{V}W^\dagger\rangle\!\rangle\langle\!\langle\hat{V}| = \sum_{V\in\mathbb{W}_{d,n}} \chi_V(W)|\hat{V}\rangle\!\rangle\langle\!\langle\hat{V}| \tag{S41}$$

where

$$\chi_V(W) = \langle\!\langle\hat{V}|W\hat{V}W^\dagger\rangle\!\rangle = D^{-1}\operatorname{tr} V^\dagger WVW^\dagger, \tag{S42}$$

which is a character of the projective Weyl group. By Schur's orthogonality relations, for any $U \in \mathbb{W}_{d,n}$ we have

$$\mathbb{E}_{W\in\mathbb{W}_{d,n}} \sum_{V\in\mathbb{W}_{d,n}} \chi_U^*(W)\chi_V(W)|\hat{V}\rangle\!\rangle\langle\!\langle\hat{V}| = |\hat{U}\rangle\!\rangle\langle\!\langle\hat{U}|, \tag{S43}$$

so that if we set $\Theta(2j, W_j) = \chi_{U_j}^*(W_j)\phi(W_j)$ for $U_j \in \mathbb{W}_{d,n}$, which can be accomplished by multiplying $\mu_{\Theta,\vec{W}}$ by $\chi_{U_j}^*(W_j)$ for each j, we find that eq. (S36) simplifies to

$$\Lambda_Q|\hat{U}_m\rangle\!\rangle\langle\!\langle\hat{U}_0|\Lambda_\psi \prod_{j=m\to 1} \langle\!\langle\hat{U}_j|\Theta(2j-1, C)|\hat{U}_{j-1}\rangle\!\rangle, \tag{S44}$$

which is now a product of scalars so we can reorder terms as desired. When C is a Clifford operator, choosing $U_j = C^jU_0C^{-j}$ for some fixed $U_0 \in \mathbb{W}_{d,n}$ reduces eq. (S44) to the expression in Ref. [8].

C. CB variance

We now analyze the variance to lowest order to show how the unitarity [9] can be estimated. The analysis closely parallels that of Ref. [9], except that because we are using a weaker twirl (namely, over the Heisenberg-Weyl group instead of over the full multi-qudit Clifford group), the matrix that governs the decay rates has more eigenvalues. As in Ref. [9], We use the fact that for any scalar λ, we have $|\lambda|^2 = \lambda \otimes \lambda^*$ and that multiplication distributes across tensor products, so that we will consider the implementation map $\Theta = \Lambda \otimes \Lambda^*$, where the phases added when analyzing a decay cancel. From eq. (S36), we then have

$$\begin{aligned} \mathbb{E}_{\vec{W}} |\mu_{\Lambda,\psi,\vec{W},Q}|^2 &= \mathbb{E}_{\vec{W}} \mu_{\Theta,\psi,\vec{W},Q} \\ &= \Theta_Q \left(\prod_{j=m\to 1} \Theta(j)\Theta(C) \right) \Theta(0)\Theta_\psi. \end{aligned} \tag{S45}$$

Applying Schur's orthogonality relations gives

$$\begin{aligned} \Theta(j) &= \mathbb{E}_{W\in\mathbb{W}_{d,n}} \phi(W) \otimes \phi^*(W) \\ &= \sum_{U,V\in\mathbb{W}_{d,n}} |\hat{U}\otimes\hat{V}\rangle\!\rangle\langle\!\langle\hat{U}\otimes\hat{V}| \mathbb{E}_{W\in\mathbb{W}_d^{\otimes n}} \chi_U(W)\chi_V^*(W) \\ &= \sum_{U,V\in\mathbb{W}_{d,n}} |\hat{U}\otimes\hat{V}\rangle\!\rangle\langle\!\langle\hat{U}\otimes\hat{V}| \delta_{U,V} \\ &= \sum_{U\in\mathbb{W}_{d,n}} |\hat{U}\otimes\hat{U}\rangle\!\rangle\langle\!\langle\hat{U}\otimes\hat{U}|. \end{aligned} \tag{S46}$$

In particular, $\Theta(j)^2 = \Theta(0)$ for all j and so eq. (S45) can be written as

$$\mathbb{E}_{\vec{W}} |\mu_{\Lambda,\psi,\vec{W},Q}|^2 = \Theta_Q M^m \Theta_\psi, \tag{S47}$$

where

$$M = \Theta(0)\Theta(C)\Theta(0) = \sum_{U,V\in\mathbb{W}_{d,n}} |\hat{U}\otimes\hat{U}\rangle\!\rangle\langle\!\langle\hat{V}\otimes\hat{V}| |\Lambda_{UV}(C)|^2. \tag{S48}$$

For simplicity, we assume that $\Lambda(C)$ is unital and trace preserving, that is, that for all $U \in \mathbb{W}_{d,n}$ we have

$$\Lambda_{U,I}(C) = \Lambda_{I,U}(C) = \delta_{I,U}. \tag{S49}$$

Then we can rewrite eq. (S47) as

$$\begin{aligned} \mathbb{E}_{\vec{W}} |\mu_{\Lambda,\psi,\vec{W},Q}|^2 &= \Theta_Q M_{\mathrm{u}}^m \Theta_\psi + \Theta_Q |\hat{I}^{\otimes 2}\rangle\!\rangle\langle\!\langle\hat{I}^{\otimes 2}|\Theta_\psi \\ &= \Theta_Q M_{\mathrm{u}}^m \Theta_\psi + |\Lambda_Q|\hat{I}\rangle\!\rangle\langle\!\langle\hat{I}|\Lambda_\psi|^2, \end{aligned} \tag{S50}$$

where we define the unital block of M to be

$$M_{\mathrm{u}} = \Theta(0)\Theta(C)\Theta(0) = \sum_{U,V\in\mathbb{W}^*_{d,n}} |\hat{U}\otimes\hat{U}\rangle\!\rangle\langle\!\langle\hat{V}\otimes\hat{V}| |\Lambda_{UV}(C)|^2 \tag{S51}$$

and $\mathbb{W}^*_{d,n} = \mathbb{W}_{d,n} - \{I\}$. Further, note that the constant term is can be directly estimated from eq. (S44) for any C by setting $U_0 = I$, and so for unital noise the variance over random Weyl operators satisfies

$$\mathbb{E}_{\vec{W}} |\mu_{\Lambda,\psi,\vec{W},Q}|^2 - |\mathbb{E}_{\vec{W}} \mu_{\Lambda,\psi,\vec{W},Q}|^2 = \Theta_Q M_{\mathrm{u}}^m \Theta_\psi. \tag{S52}$$

We now show how the unitarity of a channel [9] can be estimated from eq. (S52) under assumptions similar to those in XEB. First, note that if M_{u} is diagonalizable, eq. (S47) can be written as

$$\mathbb{E}_{\vec{W}}|\mu_{\Lambda,\vec{W}}|^2 = \sum_j \alpha_j \lambda_j^m \tag{S53}$$

where the λ_j are the nonzero eigenvalues of M_{u} and the α_j are the overlaps of the corresponding eigenvectors of M_{u} with the SPAM vectors. We thus want to prove that the unitarity of a channel corresponds to an eigenvalue of M_{u}. The unitarity u of a quantum channel is defined to be [9]

$$u^2 = \frac{1}{D^2-1} \sum_{U,V\in\mathbb{W}^*_{d,n}} |\Lambda_{UV}(C)|^2. \tag{S54}$$

As in XEB, we now assume that the error model consists only of a unitary error and global depolarization, so that

$$\Lambda(C) = \phi(T)\mathcal{D}_p \tag{S55}$$

for some unknown $T \in \mathbb{U}(D)$ where

$$\mathcal{D}_p(\rho) = p\rho + (1-p)I/D \tag{S56}$$

is the global depolarizing channel. Under this assumption, we have $u = p$, so that the goal is to learn an effective depolarizing rate of the noise process. We now show that

$$v = \sum_{U\in\mathbb{W}^*_{d,n}} |\hat{U}^{\otimes 2}\rangle\!\rangle \tag{S57}$$

is an eigenvector of M_{u} with eigenvalue p^2. First note that for all $U \in \mathbb{W}^*_{d,n}$, we have

$$\sum_{V\in\mathbb{W}^*_{d,n}} |\Lambda_{U,V}|^2 = p^2. \tag{S58}$$

Therefore

$$\begin{aligned} M_{\mathrm{u}}v &= \sum_{U,V,W\in\mathbb{W}^*_{d,n}} |\hat{U}^{\otimes 2}\rangle\!\rangle |\Lambda_{U,V}(C)|^2 \langle\!\langle \hat{V}^{\otimes 2}|\hat{W}^{\otimes 2}\rangle\!\rangle \\ &= \sum_{U\in\mathbb{W}^*_{d,n}} |\hat{U}^{\otimes 2}\rangle\!\rangle p^2 \\ &= p^2 v \end{aligned} \tag{S59}$$

that is, we have an eigenvector of M_{u} the purity matrix whose eigenvector is exactly p^2. Moreover, by eq. (S58), M_{u}/p^2 is a stochastic matrix and so its largest eigenvalue is 1. Therefore, under the assumption of global depolarizing and unitary noise, the largest eigenvalue of the unital part of M_{u} is u^2.

We now show how we can estimate the eigenvalues of M_{u}. Let s be the order of C, that is, the smallest positive integer such that $C^s \propto I$ and assume that s divides m. Then in the limit of high fidelity, the eigenvectors of M_{u}^s are $|\hat{U}^{\otimes 2}\rangle\!\rangle$, and so we can find the largest eigenvalue of M_{u} (and hence the purity under the assumption of global depolarizing and unitary noise) by finding the slowest decay rate of eq. (S52).

Supplementary Note 7 . QUTRIT CZ GATE BENCHMARKING

Here we provide the cycle benchmarking results of the 783 ns qutrit CZ gate from the main text. As with the $\mathrm{CZ}^\dagger$, we estimate the process fidelity of the isolated CZ gate by comparing our CB results to a reference cycle, and using the formula:

$$e_F = \frac{D-1}{D}\left(1 - \frac{\mathcal{F}_{\mathrm{CZ}}}{\mathcal{F}_{\mathrm{Reference}}}\right) \tag{S60}$$

where D is the dimension of the Hilbert space of one's system. In our benchmarking of the CZ, we find a Weyl infidelity of 0.0861 for the dressed CZ cycle and 0.034 for the reference cycle. Ultimately isolating the errors of the CZ gate, we find an estimated process fidelity of 95.2(3)%.

‘

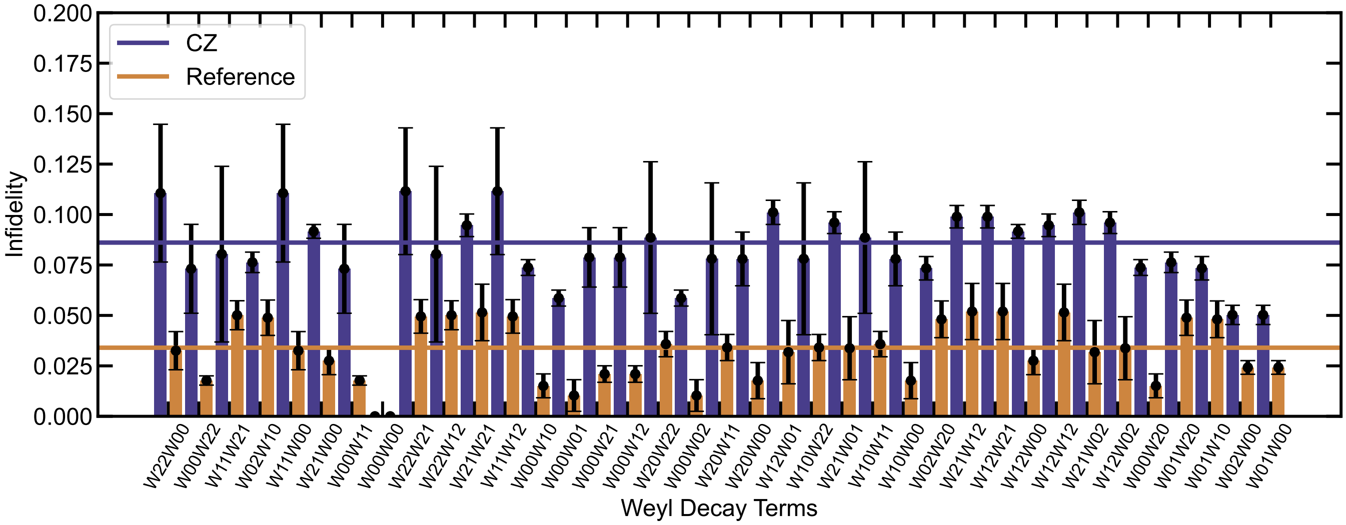

Supplementary Fig. 4. **Cycle benchmarking results with Weyl decay terms of the qutrit CZ gate**. The cycle benchmarking results analyze a subset of 54 Weyl channels at depths $m \in \{0, 3, 6\}$ for the CZ cycle. In order to provide a SPAM free estimation of the error, we again compare our CB results to a reference CB experiment. Here we plot only the shared decay channels analyzed for both the CZ and reference CB experiments. The error bars are due to the uncertainty in the fitting of each decay.

Supplementary Note 8 . FREQUENCY DEPENDENCE OF DRIVEN CROSS-KERR

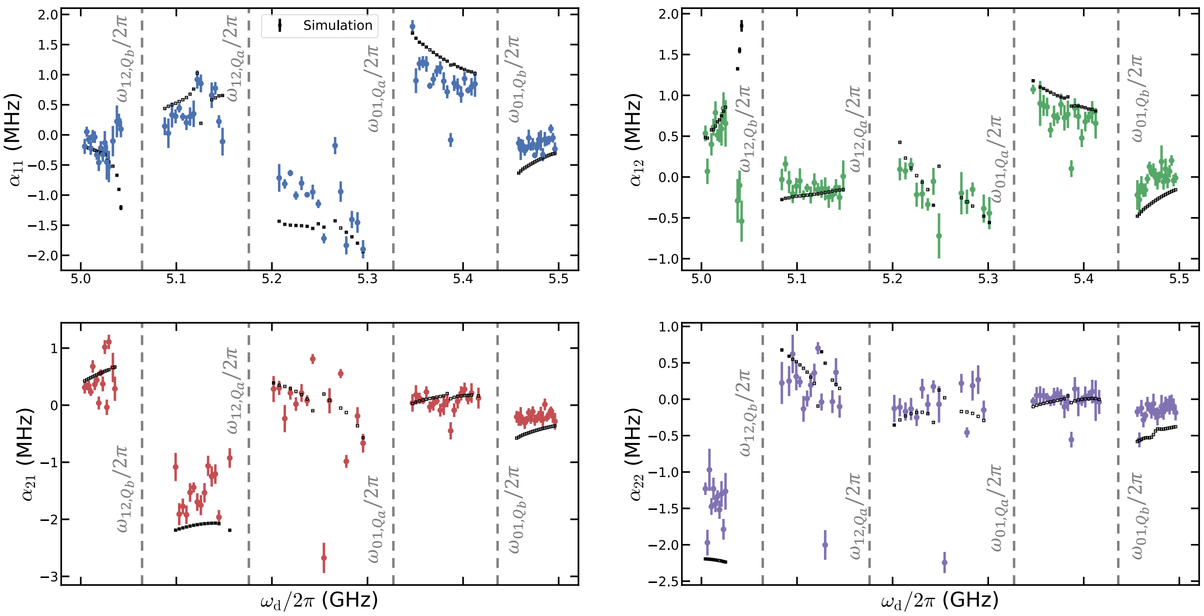

Supplementary Fig. 5. **Frequency dependence of the full microwave-activated cross-Kerr Hamiltonian**. We compare the dependence of all of the α_{ij} in the driven cross-Kerr Hamiltonian on the frequency of the drive ω_d using an *ab-initio* master equation simulation in QuTiP. The error bars are due to the uncertainty in each linear fit of the entangling phase.

In Figure 2 in the main text, we showed the results of fitting the frequency dependence of the α_{12} term in the driven cross-Kerr Hamiltonian to an *ab-initio* master equation simulation. Here we provide some additional details on how this simulation was performed in QuTiP [10, 11] and present the results for measuring and characterizing the frequency dependence of all four of the α_{ij} in the driven cross-Kerr Hamiltonian. We note that transient TLS features and higher transitions meant that some of the data did not fit to a linear model, we therefore only plot data where the uncertainty on our linear fit (the source of the error bars) was less than 300 KHz. Similarly, our simulation at times produced unphysical results near transitions, with very large cross-Kerr; in the interest of readability, we therefore also omitted points where the magnitude of the simulated cross-Kerr was larger than 3 MHz.

To perform the master equation simulation, we considered the Hamiltonian of two fixed frequency transmons, with a fixed capacitive coupling from a coplanar waveguide resonator. For the frequencies and anharmonicities of the pair of transmons, we used the experimental parameters from our chip (as can be found in Table 1). We first found the strength of the capacitive coupling, J, by adjusting it until the simulated parameters for the always on, static α_{ij} best matched our experimental measurements of these parameters. We then fixed a single drive frequency, ω_d, and performed a master equation simulation of the simultaneous Stark drives at that frequency until our data best matched the values found for that point in Supplementary Fig. 5. After this, we extrapolated those parameters to be the same across all frequencies of the Stark driving, and simulate using them for the rest of the frequencies in Supplementary Fig. 5.

Supplementary Note 9 . QUANTUM PROCESS TOMOGRAPHY

We analyze the Pauli transfer matrix (PTM) of the $CZ^\dagger$ gate in the Gell-Mann basis. To construct the PTM in this basis, we prepare 81 two-qutrit input states by applying an informationally complete set of native gates on each qutrit: $\{I, \mathrm{X}_{\pi/2}^{(01)}, \mathrm{Y}_{\pi/2}^{(01)}, \mathrm{X}_{\pi}^{(01)}, \mathrm{X}_{\pi}^{(12)}\mathrm{X}_{\pi}^{(01)}, \mathrm{Y}_{\pi}^{(12)}\mathrm{X}_{\pi/2}^{(01)}, \mathrm{X}_{\pi/2}^{(12)}\mathrm{X}_{\pi}^{(01)}, \mathrm{Y}_{\pi/2}^{(12)}\mathrm{X}_{\pi}^{(01)}, \mathrm{X}_{\pi}^{(12)}\mathrm{X}_{\pi/2}^{(01)}\}$. We measure the output tomography using the same set of native gets, and reconstruct the PTM from the data using maximum likelihood estimation method. Supplementary Fig. 6 shows the reconstructed PTM of the $CZ^\dagger$ gate $\mathcal{E}_{\mathrm{exp}}$ in the Gell-Mann basis, and in Supplementary Fig. 7 we plot $\mathcal{E}_{\mathrm{ideal}}^\dagger \mathcal{E}_{\mathrm{exp}}$. The process fidelity is calculated from the PTM to be $\mathcal{F}_{\mathrm{PTM}} = \mathrm{Tr}[\mathcal{E}_{\mathrm{ideal}}^\dagger \mathcal{E}_{\mathrm{exp}}]/D^2 = 93.2\%$. We note that as process tomography does not decouple our characterization of our gate errors from state preparation and measurement errors, it is disfavored as a benchmarking technique when compared to CB or XEB.

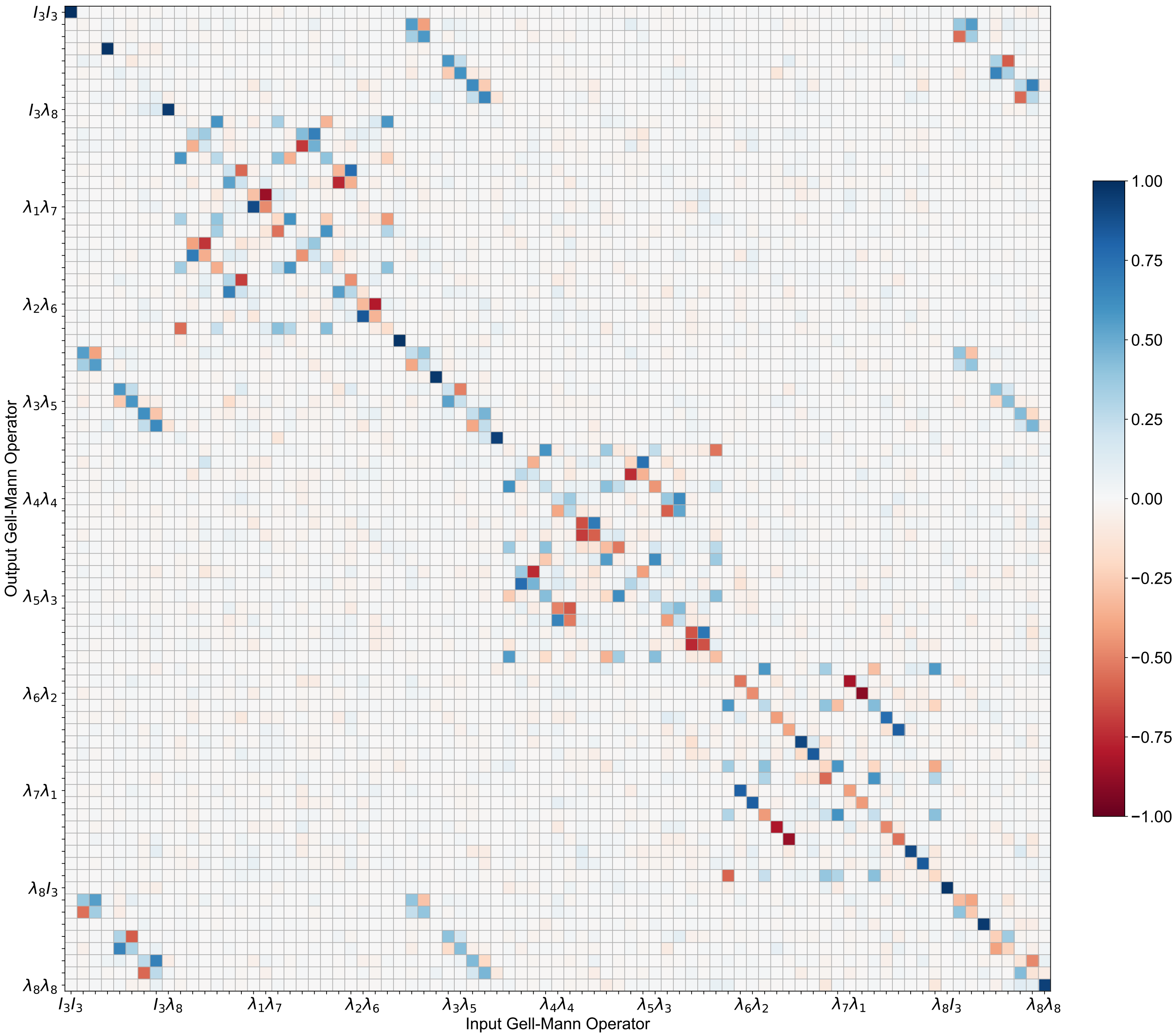

Supplementary Fig. 6. **CZ† process matrix**. Experimentally reconstructed Process Matrix of Qutrit CZ†($\mathcal{E}_{\text{exp}}$) gate with process fidelity of 93.2%.

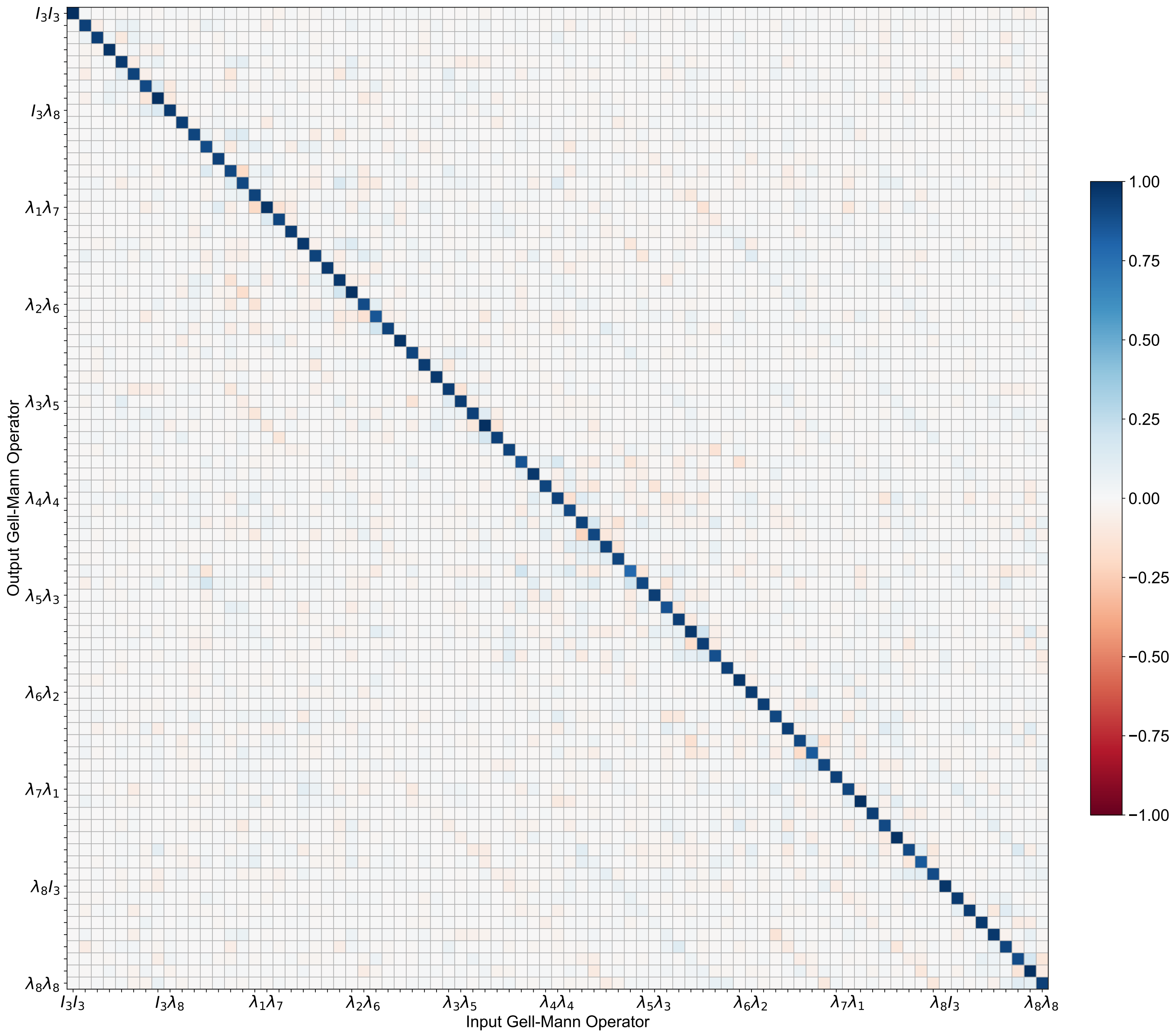

Supplementary Fig. 7. **Comparing the experimentally reconstructed process matrix to the ideal case**. We plot $\mathcal{E}_{\rm ideal}^{\dagger}\mathcal{E}_{\rm exp}$, from which we can estimate the process fidelity of the CZ† gate.

[1] Mitchell, B. K. *et al.* Hardware-efficient microwave-activated tunable coupling between superconducting qubits. *Phys. Rev. Lett.* **127**, 200502 (2021). URL https://link.aps.org/doi/10.1103/PhysRevLett.127.200502.
[2] Morvan, A. *et al.* Qutrit randomized benchmarking. *Phys. Rev. Lett.* **126**, 210504 (2021). URL https://link.aps.org/doi/10.1103/PhysRevLett.126.210504.
[3] Helsen, J., Roth, I., Onorati, E., Werner, A. H. & Eisert, J. A general framework for randomized benchmarking. *arXiv* (2020).
[4] Arute, F. *et al.* Quantum supremacy using a programmable superconducting processor. *Nature* **574**, 505–510 (2019). URL https://doi.org/10.1038/s41586-019-1666-5.
[5] Kim, Y. *et al.* High-fidelity three-qubit itoffoli gate for fixed-frequency superconducting qubits. *Nature Physics* (2022). URL https://doi.org/10.1038/s41567-022-01590-3.
[6] Mullane, S. Sampling random quantum circuits: a pedestrian's guide (2020). arXiv:2007.07872.
[7] Dita, P. Factorization of unitary matrices. *Journal of Physics A: Mathematical and General* **36**, 2781–2789 (2003). URL https://doi.org/10.1088/0305-4470/36/11/309.
[8] Erhard, A. *et al.* Characterizing large-scale quantum computers via cycle benchmarking. *Nature Communications* **10**, 5347 (2019). URL https://doi.org/10.1038/s41467-019-13068-7.
[9] Wallman, J., Granade, C., Harper, R. & Flammia, S. T. Estimating the coherence of noise. *New Journal of Physics* **17**, 113020 (2015). URL https://doi.org/10.1088/1367-2630/17/11/113020.
[10] Johansson, J., Nation, P. & Nori, F. Qutip: An open-source python framework for the dynamics of open quantum systems. *Computer Physics Communications* **183**, 1760–1772 (2012). URL https://www.sciencedirect.com/science/article/pii/S0010465512000835.
[11] Johansson, J., Nation, P. & Nori, F. Qutip 2: A python framework for the dynamics of open quantum systems. *Computer Physics Communications* **184**, 1234–1240 (2013). URL https://www.sciencedirect.com/science/article/pii/S0010465512003955.
